# Spike-microstates correlate with interictal epileptogenic discharges: a marker for hidden epileptic activity

**DOI:** 10.1093/braincomms/fcad124

**Published:** 2023-04-18

**Authors:** Vincent Rochas, Markus Gschwind, Krassen Nedeltchev, Margitta Seeck

**Affiliations:** EEG and Epilepsy Unit, Department of Neurology University Hospital Geneva and University of Geneva, 1201 Geneva, Switzerland; EEG and Epilepsy Unit, Department of Neurology University Hospital Geneva and University of Geneva, 1201 Geneva, Switzerland; Department of Neurology, Kantonsspital Aarau, 5001 Aarau, Switzerland; Department of Neurology, Kantonsspital Aarau, 5001 Aarau, Switzerland; EEG and Epilepsy Unit, Department of Neurology University Hospital Geneva and University of Geneva, 1201 Geneva, Switzerland

**Keywords:** epilepsy, EEG, biomarker, microstates, pre-surgical evaluation

## Abstract

Objectively estimating disease severity and treatment success is a main problem in outpatient managing of epilepsy. Self-reported seizures diaries are well-known to underestimate the actual seizure count, and repeated EEGs might not show interictal epileptiform discharges (IEDs), although patients suffer from seizures. In this prospective study, we investigate the potential of microstate analysis to monitor epilepsy patients independently of their IED count.

From our databank of candidates for epilepsy surgery, we included 18 patients who underwent controlled resting EEG sessions (with eyes closed, 30 min), at around the same time of the day, during at least four days (range: 4–8 days; mean: 5). Nine patients with temporal foci, six with extratemporal foci, and three with generalized epilepsy were included. Each patient’s IEDs were marked and the topographic voltage maps of the IED peaks were averaged, and an individual average spike topography (AST) was created. The AST was then backfitted to each timepoint of the whole EEG resulting in the Spike-Microstate (SMS). The presence of the SMS in the residual EEG outside of the short IEDs epochs was determined for each recording session in each patient and correlated with the occurrence of the IEDs across all recording session, as well as with the drug charge of each day.

Overall, SMS was much more represented in the routine EEG than the IEDs: they were identified 262 times more often than IEDs. The SMS time coverage correlated significantly with the IED occurrence rate (rho = 0.56; *P* < 0.001). If only patients with focal epilepsy were considered, this correlation was even higher rho = 0.69 (*P* < 0.001). Drug charge per day did not correlate with SMS.

In this proof-of-concept study, the time coverage of SMS correlated strongly with the occurrence rate of the IEDs, they can be retrieved in the scalp EEG at a much higher occurrence rate. We conclude that SMS, once obtained for a given patient, are a more abundant marker of hidden epileptic activity than IEDs, in particular in focal epilepsy, and can be used also in absence of IEDs. Future larger studies are needed to verify its potential as monitoring tool and to determine cut-off values when drug protection becomes imperfect.

## Teaser

In candidate epilepsy-surgery patients, Rochas *et al*. investigate the occurrence of the EEG topographical voltage map derived from the patient's specific epileptiform discharges. Their time course (Spike-Microstate) in EEG along several days shows to be a more abundant marker of disease activity, even in absence of countable epileptiform discharges.

## Graphical Abstract

**Graphical abstract:**
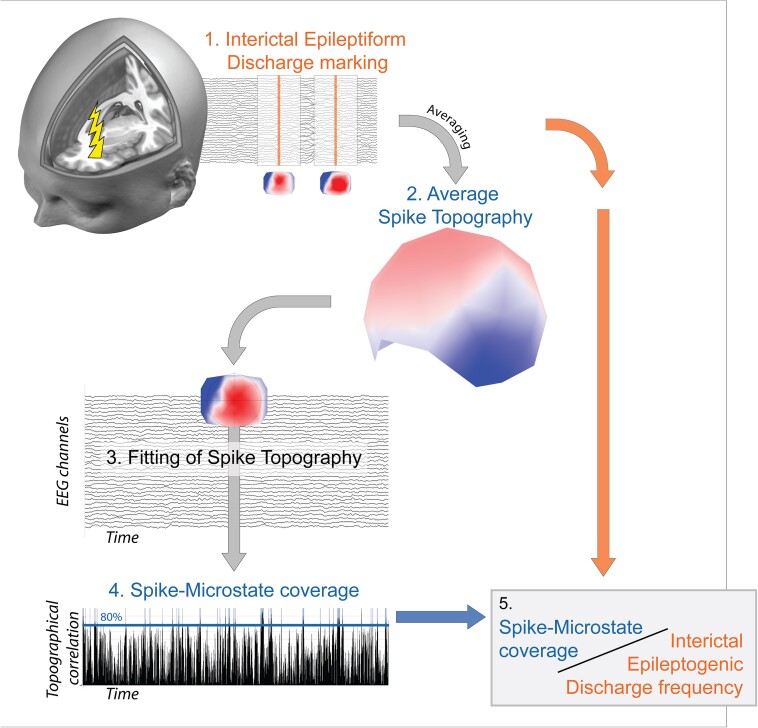

## Introduction

In outpatient monitoring of epilepsy, a realistic estimation of the patient’s disease activity is crucial. Since the milestone study of Cook *et al*. comparing the patient-reported seizure count with the intracranially recorded seizures, it is well established that there are unfortunately major discrepancies between both counts. Patients mainly tend to underreport their seizures, but overreporting was also noted.^1,2^ Many factors interfere with patients’ correct reports on seizure occurrence, for example patients may be amnesic for seizures, even for tonic–clonic seizures, in up to 30% of focal seizures,^3^ or voluntarily underreport the count (e.g. to keep the driving license), or simply forget to note and share the seizures with the treating physician during the appointment. The impossibility to obtain reliable seizure counts is a major handicap for establishing efficacious clinical patient monitoring of anti-seizure therapy.

There is an ongoing research on objective markers of seizure activity in the interictal period, which for evident reasons should be based on routine clinical EEG.^4^ Interictal epileptiform discharges (IEDs) are probably among the most established surrogate markers of seizure activity. Although a clear linear relationship with increased seizure risk has not been shown in previous studies on routine EEGs, recent ultradien recordings during several months and years from implanted electrodes suggested that just before, during or after a surge of IED, seizure risk is largest.^5^ While these findings support the connotation of IEDs as a sign of seizure activity, more tools are needed that allow the objective estimation of instant seizure risk from routine scalp EEGs.

In a previous study, Grouiller *et al*. showed in epilepsy surgery candidates that the ‘spike’-specific EEG voltage map (using averaged IEDs recorded during presurgical long-term EEG monitoring) correlated highly with the hemodynamic changes in the functional magnetic resonance imaging (fMRI), and were spatially concordant with epileptic focus from intracranial EEG or with successful resection area. It is of interest that the localizing information in the fMRI was obtained solely with spike voltage maps in absence of IEDs during EEG recording inside the scanner.^6^

Inspired from this result, we were interested to determine if the IED-derived voltage maps are also present when no IED was visible in the EEG.^7^ In the canonical approach, microstates were described as a sequence of all quasi-stable voltage topographies in the ongoing EEG during periods of high global field power (GFP).^8–10^ Its promise to detect altered brain dynamics with scalp EEG has been shown for several brain diseases over the last 20 years, such Lewy-Body disease,^11^ Alzheimer’s disease,^12–14^ multiple sclerosis,^15^ clinically manifest schizophrenia,^16–18^ as well as at risk for schizophrenia.^19–21^ The epilepsy-specific voltage topography of the patient’s IED has been used as a substrate for electric source localization,^22,23^ but also as a marker for the patient’s subtle ongoing epileptic activity (EA) in absence of IEDs.^24^

In our approach, the patient’s averaged topographic map of his own epilepsy-specific IED is computed and taken as a-priori information to search for its appearance across the whole EEG track, which corresponds to the ‘Spike-Microstates’ (SMSs). We hypothesize that these SMS correlate with the frequency of the IEDs and consequently that the presence of SMS should increase after drug withdrawal over the daily EEG recordings in patients hospitalized for presurgical monitoring long-term EEG.

## Method

### Patients

We reviewed our databank of presurgical candidates who were evaluated in our center. Inclusion criteria were unifocal or idiopathic generalized epilepsy (IGE) as determined by the presurgical work-up including interictal and ictal EEG as well as seizure semiology, with controlled resting EEG recording at least on four different days. In patients with presumed unifocal epilepsy, the patient’s MRI was reviewed by an expert neuroradiologist and in case, no lesion could be found, information from PET, ictal single-photon emission computed tomography, and MR-based structural morphometry was used to determine the epilepsy type or syndrome^25–27^ together with the interictal and ictal EEG. The diagnosis of IGE required characteristic generalized ictal and interictal discharges (generalized 3–4 Hz spike wave complexes), absence of MRI lesion and a normal PET metabolism. Exclusion criteria were the presence of multifocal epilepsy, multifocal lesions, previous operation and progressive non-epileptic brain disease, no recording or too little time of resting EEGs in a controlled condition.

### Drug monitoring

In order to study the possible effect of drug withdrawal on IEDs, we translated drug changes during the days of recording of the presurgical evaluation to a score of drug load. Facing a very diverse picture of molecules and their half-life, both between and within patients, we calculated an ordinal score reflecting drug increases or decreases of the entire medication (without hierarchy between molecules). Any dose decrease of at least one drug, including suspension of one or all drugs, compared to baseline on admission was coded as -1, any dose increase of at last one drug as +1 and 0 when there was no change to the day before.

### EEG recording and spike identification

During monitoring, each patient was recorded continuously with 38 channel system including ECG and scalp electrodes using FCz as reference and a sampling rate of 256 Hz. We recorded 30 min of resting EEG each day between 14 and17 h, during which patients were asked to close the eyes and simply rest without falling asleep. All patients were in single rooms, and the environment was kept quiet. The recordings were obtained on a daily basis, but for logistical reasons, only during weekdays, and if no exams were scheduled and no seizure occurred.

The EEG was band pass filtered from 1 to 70 Hz, and the periods of significant artefact were rejected from recordings. An additional 50 Hz notch filtering was applied. An independent component analysis decomposition of each EEG recording was performed and heart and muscle movements’ components were removed.

### Marking interictal epileptogenic discharges

**Figure 1:**
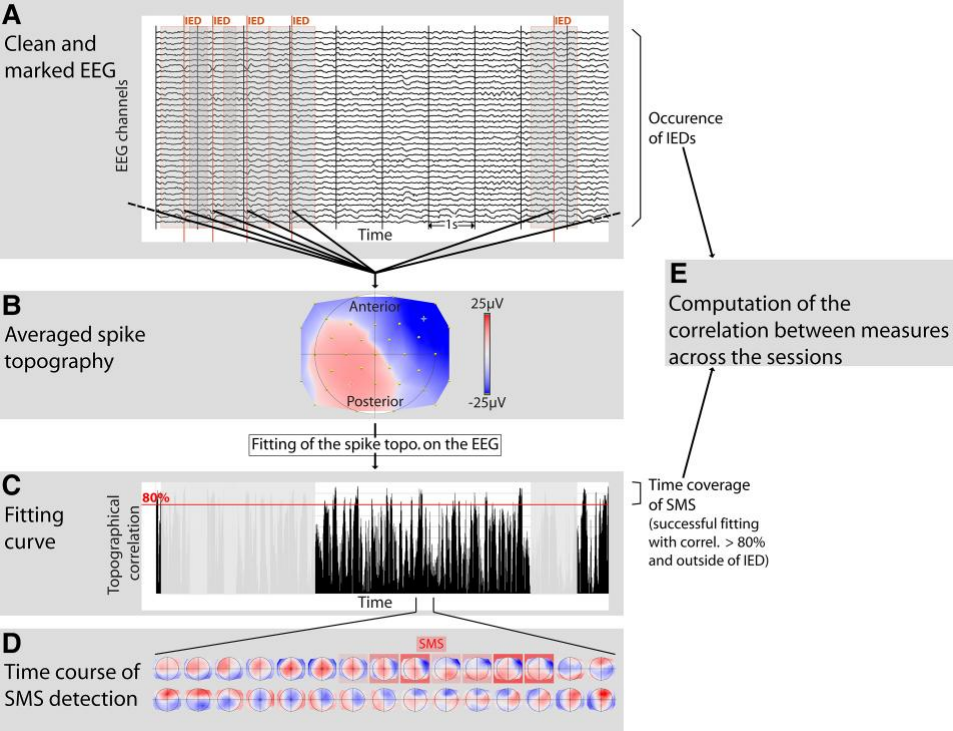
**The different steps of the spike-microstate analysis in one example patient.** (**A**) the IEDs are marked in the clean EEG, red line, and a period of +/- 500 ms around the IED is marked as exclusion time for latter SMS detection (highlighted grey bands). (**B**) the IED are averaged across the recordings of the sessions for every patient and the averaged spike topography (AST) is extracted as an individual template. (**C**) the resulting topographic template is back topographically fitted to the whole session EEG in order to characterise (**D**) the Spike-Microstate (SMS) time coverage (highlighted red squares). (**E**) Both measures of IEDs and SMS are put in comparison for further analyses.

IEDs were retrieved by one of us (VR) who was blinded to the clinical history of the patients (see Fig. 1A). IEDs included spikes, spike-waves, polyspikes, polyspike-waves, sharp waves, and sharp-slow wave complexes. An automatic spike detection was first performed using Persyst® software, and then carefully reviewed to verify correct labelling of IEDs. This allowed adding missed spikes and removing false alarms. Two patients were excluded from the study due to total lack of IED during their resting recordings (one had only IED during night sleep, and one was suspected to have psychogenic seizures). Hence, further analyses are continued on 18 patients (seven females, median age: 39.5, range: 15–58).

IEDs were time locked at the moment of maximum GFP (i.e. the spike peak), and subsequently averaged for each channel. The averaged spike peak was used as a template EEG topography—called average spike topography (AST) (see Fig. 1B).

### Microstate computation

Using the software Cartool, the AST was backfitted to the filtered and artefact-removed EEG recordings of all different sessions of the patient. This fitting consisted in the computation of the spatial correlation of the AST with the instant topography at each time point of the EEG. It was performed using average reference, after a normalisation of the EEG by the GFP,^9^ ignoring the topography polarity, and applying a spatial smoothing filter but no temporal smoothing. This fitting procedure provided therefore a correlation curve (from 0 to 1) which is an index of the fitting strength between the AST and the whole EEG at each time point, or in other words, how well the topography of the spike peak is representative of the EEG across time.

### Statistical analysis

At the time points of the IEDs, the correlation value is expected to be maximal with the SMS. To avoid this confound, we discarded the short period around the IEDs from fitting, i.e. from 500 ms before the IED to 500 ms after the IED, so that only EEG independent from IEDs themselves was subject to analysis. In order to keep only the most relevant time points of the fitting, we chose a threshold for the fitting correlation value above 0.8 which is considered as successful fitting and represented the ‘SMSs.’ The presence of SMS was calculated for each session by dividing the number of time frames containing SMS by the total number of scanned time frames, defined as time coverage. Similarly, the IED occurrence was calculated for each session as the number of IEDs divided by the total number of scanned time frames. Hence each session had a specific value for SMS and IED representation.

For exploratory purpose, we first computed Pearson correlation coefficient *R* between both SMS and IEDs measures across all sessions for each patient. In a second step using the software SPSS 25, a linear mixed model with multiple sessions as repeated measure was computed for fixed effects with the occurrence of IEDs as dependent variable, the drug score as explicative factor and coverage of SMS as covariable.

### Data availability

At the time of data acquisition, public data sharing was not requested in our ethics committee application, but data can be made available to interested research groups upon request.

## Results

Eighteen patients (seven females) matched our inclusion criteria and were considered in the present study (Table 1). Nine patients suffered from temporal lobe epilepsy, six from extra-temporal lobe epilepsy, and three from IGE. The mean age at evaluation (range 14–57) was 34.7 years (+/- 15.7) and age of onset 21.0 (+/- 15.8). Patients were hospitalized on average during nine days. We obtained 30 min daily recordings on four to eight different days per patients (mean: five days).

**Table 1:** Patient characteristics

	Sex	Age	Age at onset (y)	Epilepsy duration (y)	MRI	Epilepsy syndrome (ictal and interictal EEG)	Count of interictal discharges
1	M	57	53	4	N	R temporal	696
2	F	40	23	17	N	L anterior temporal	378
3	M	47	10	37	N	R frontal	1047
4	M	25	13	12	N	IGE	334
5	M	48	8	40	R frontal dysplasia	R frontal	1539
6	M	14	57	43	L Hippocampal sclerosis	L temporal	1230
7	M	36	16	20	N	IGE (initially left frontal IEDs)	99
8	F	58	34	24	L parietal cavernoma	L frontal	12
9	M	23	18	5	N	R frontal	28
10	F	57	36	21	N	R temporal	830
11	F	45	14	31	R temporal DNET	R temporal	405
12	F	48	22	26	N	R temporal	263
13	F	16	10	6	N	L parietal	1519
14	M	40	34	6	R temporal dysplasia	R temporal	757
15	F	20	6	14	N	IGE	188
16	M	15	3 months	15	L parieto-occipital dysplasia	L parietal	2121
17	M	36	1	35	L hippocampal sclerosis	L temporal	2065
18	M	39	23	16	Post-traumatic with largest lesion in the L temporal lobe	L temporal	627

Abbreviations: M = male, F = female; N = normal, L = left, R = right, DNET = dysembryoplastic neuroepithelial tumor; IGE = idiopathic generalized epilepsy.

The average drug score across sessions and patients was -0.36, reflecting the drug decrease during presurgical evaluation.

**Figure 2:**
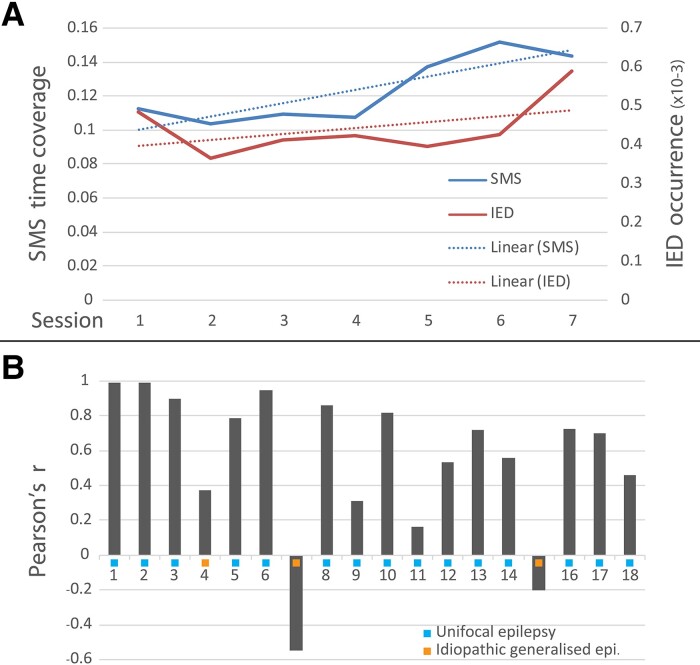
**Spike-Microstate correlation with interictal epileptiform discharges**. (**A**) Evolution of the averages across the 18 included patients for occurrences of IED (red line) and SMS (blue line) as ratio of time across sessions and their linear trends (dashed lines). Linear mixed model showed a significant correlation of time coverage of SMS as the explicative covariable on the occurrence of IEDs (*F* = 8.43; Sig. = 0.004). (**B**) Individual coefficients of Pearson correlation between occurrence of IED and time coverage of SMS for the 18 included patients. The light blue squares show unifocal, the orange squares show idiopathic generalised epilepsy types.

The average IED occurrence for the whole group was 4.2 × 10^−4^, ranging from 1.1 × 10^−5^ to 1.7 × 10^−3^ per patient. The average SMS time coverage was 0.11, ranging from 0.03 to 0.29 per patient. As a result, SMS was 262 times more present than IEDs. On average, both were increasing along the days of monitoring after drug withdrawal (see Fig. 2A). The individual correlations between IEDs and SMS measures were defined with an average coefficient of rho = 0.56, and positive in all except two patients (Fig. 2B). If only patients with unifocal epilepsy were considered (*N* = 14), the average correlation was rho = 0.69.

Across all subjects (*N* = 18), the linear mixed model with the occurrence of IEDs as dependent variable and time coverage of SMS as the explicative covariable showed a highly significant correlation (*F* = 8.43; Sig. = 0.004; CI from 0.000701 to 0.003697). However, there was no effect of the drug score explaining the IEDs (*F* = 0.39; Sig. = 0.76; CI from -0.000236 to 0.000471). If only patients with unifocal epilepsy are considered (*N* = 14), the linear mixed model showed an even stronger significant correlation of SMS on IEDs (*F* = 24.30; Sig. = 4 × 10^−6^; CI from 0.001893 to 0.004453), but again no effect of the drug load (*F* = 0.20; Sig. = 0.90; CI from -0.000233 to 0.000308).

The three patients with IGE had lower or negative correlations between IEDs and SMS (patients 4, 7, 15, see Fig. 2B). Interestingly, the IGE-patient 4 with a correlation of 0.37 initially had many left frontal spikes, being the reason for the referral to presurgical work-up. However, after drug withdrawal, he presented primary generalized seizures with typical generalized IEDs in the EEG. After introducing Valproate therapy, no more IEDs and no seizures were recorded.

We were interested if the exclusion or inclusion of the EEG around the IEDs change the results in a considerable way. Therefore, we recomputed the same analyses, now calculated on the whole EEGs. The results were similar, showing a positive correlation between IEDs and SMS occurrences with an average coefficient of rho = 0.59, while the linear mixed model on IED occurrence showed a highly significant effect of the covariable SMS occurrence (*F* = 10.62; Sig. = 0.001; CI from 0.000882 to 0.003560). Again, the drug load effect was not significant (*F* = 0.39; Sig. = 0.76; CI from -0.000232 to 0.000474).

This only minimal difference between a calculation excluding the IED epochs and a calculation including the IEDs epochs showed that the few points in common between SMS and IED hardly played a role, and could not inflate the correlation, because the density of SMS is in average more than 200 times higher than the density of IEDs.

## Discussion

Our study suggests that SMS related to the patients’ IEDs are more comprehensive markers of EA than visible IEDs. The high correlation of SMSs with visible IEDs strongly suggests that they both reflect the same process, particularly in patients with focal epilepsy. Several studies using simultaneous scalp and intracranial recordings showed that only a small fraction of spikes become visible on the scalp^28^ which require as much as 10–20 cm^2^ of cortical surface to be tracked.^29^ Our results suggest that we have a promising marker which corresponds more likely to the true intracranial EA.

From a practical point of view, the microstate computation is quite resilient towards EMG artefacts, as it relies on the whole topography and not on a single noisy signal. The microstate method has also been demonstrated its practicability in 4–8-year-old children.^30^ In terms of computation time, our approach is also very reasonable. The correlation values between IEDs and SMS are similar and equally high with or without marking and separating IEDs from the rest of the EEG. In the context of the study, we carried out manual artefact removal and semi-automatic marking of the IEDs, which added approximately one hour of additional work for an EEG session of 30 min. However, in a clinical perspective, the computation time would include only AST calculation and backfitting to the EEG (discarding artefact periods) and would last less than 2 min which is easily implemented in clinical practice.

The strong relationship between visible IEDs and SMS has been found only for patients with focal epilepsy. The two patients with the lowest correlations between IEDs and SMS were patients with IGE and the overall average of correlation between IEDs and SMS is lower than for patients with focal epilepsy. Thus, our approach appears to be less useful for this patient group.

It is noteworthy that the average SMS time coverage is markedly higher than the IED frequency. In other words, for one IED we observe 262 SMS on average which makes SMS an excellent candidate for a sensible marker compared to the IEDs themselves. Thus, visible IEDs appear to be the tip of the iceberg of EA, embedded in an EEG with many more SMS. Interestingly, while both parameters tend to increase with drug withdrawal, the SMS coverage increased before the IED occurrence (see Fig. 2A). Whereas general antiepileptic drug load decrease would be a reasonable explanation to the IEDs and SMS increase along the recording sessions, it could not explain the day-by-day fluctuations in our study. Interestingly, the SMS to IEDs relation remains very strong and stable despite the different application forms and doses of drugs.

The choice of 80% as a threshold for fitting considered as matching was used successfully in classical microstate analysis of resting states. Taking a threshold at 80% correlation for a successful fitting means that only the best 20% are kept. It is based on clustering decomposition of the EEG sort in between four^9,31^ and six maps,^32,33^ that are generally competitively back-fitted. In the present study, the fitting of the individual IED peak topography is by definition not a competitive fitting between resting state maps. Using a lower correlation threshold, weak and possibly non-sense correlations corresponding to a fitting by default would be taken into account. Reversely, a too high threshold would have result in identifying only segments close to the actual IEDs themselves. Furthermore, the 80/20% ratio is also a more generally accepted rule known as the Pareto principle.^31^

Our study has several limitations. The study group is small, however, it contains abundant data material from four to eight recording sessions per patient of 30 min at a sampling rate of 256 Hz, so around 460’800 time points per session were analysed. Thus, we feel that our results qualify for a proof-of-concept study. In order to minimize the effect of diurnal fluctuations of IEDs, we recorded our patients around the same time of the day. This is logistically difficult in the clinical environment but we succeeded with the help of a committed technician team. We let the patient rest with eyes closed for 30 min, which sometimes elicited light sleep and may have contributed to the appearance of IED and SMS, and consequently to a high correlation. However, if such a procedure is crucial for monitoring, it could be implemented into clinical practice and might turn out to be more useful than hyperventilation or photic stimulation in patients with focal epilepsy. We did not find a correlation with our drug load score. Most likely, this was due to large differences in drug half-life, as well as polytherapy with individual reduction schemata. Finally, the study was carried out in patients with chronic epilepsy, which may differ from early onset focal epilepsy. Future studies will show if the microstate approach is also useful at an early stage of the disease and if it differentiates between patients with chronic epilepsy, amenable for surgery, versus pharmacosensitive epilepsy.

In conclusion, we propose SMS as additional biomarker in patients with diagnosed unifocal epilepsy. Our approach requires that the patient’s IEDs be recorded at some point and characterized. Patients who never displayed IED, but only seizures, cannot be monitored with this technique. However, the computation of the SMS presence has shown not to be impacted by the inclusion or exclusion of the IED periods themselves, which means that this step of the computation could be shortcut in a potential clinical application. In this context, the identification of changes of the basic canonical microstates, which are present in the resting EEG of everybody, might be of interest in addition to the epileptogenic activity. For example, in subjects with multiple sclerosis, the duration of some of those maps are altered and reflect disease activity, despite only little neurological impairment,^15^ and microstates also showed to reflect successful neuroleptic medication.^34^ Our study adds to the body of evidence that advanced EEG analysis is helpful for patient monitoring.

## Abbreviations

AST =: average spike topography
CI =: confidence interval
EA =: epileptic activity
ETLE =: extra-temporal lobe epilepsy
IED =: interictal epileptiform discharges
IGE =: idiopathic generalized epilepsy
SMS =: Spike-Microstate
TLE =: temporal lobe epilepsy
